# Overexpression of C19orf48 correlates with poor prognosis in breast cancer

**DOI:** 10.4314/ahs.v23i2.31

**Published:** 2023-06

**Authors:** Jia Guo, Beina Hui, Tuotuo Gong, Xu Zhao, Jing Li

**Affiliations:** Department of Radiation Oncology, The First Affiliated Hospital of Xi'an Jiaotong University, Xi'an, 710061, China

**Keywords:** Breast cancer, c19orf48, Predictive biomarker

## Abstract

As one of the most commonly diagnosed cancers in women around the world, breast cancer has been detailed studied. This study aimed to identify the expression of c19orf48 in several kinds of cancers including liver, lung and breast cancers etc. The driving factors behind it were analysed and it found that the amplification of c19orf48 may relate with the elevated expression. At the same time, the correlation between the expression of it and the survival time in breast cancer patients was explored. It was found that the c19orf48 expression at transcriptional level elevated in breast cancer tissue samples compared with the normal. It was inferred that the c19orf48 play its oncogenic role in development of breast cancer by involving in cell-cycle related biological process. In conclusion, c19orf48 may be a useful and predictive biomarker for the prognosis of breast cancer patients. To the best of our knowledge, this is the first report describing the expression of c19orf48, the potential driving factor led to this and its effect.

## Introduction

Breast cancer, which is one of the most commonly diagnosed cancers in women around the world, has been extensively studied 1. Statistics from ACS indicated that there are over 1.676 billion new breast cancers and 521,900 breast cancer deaths in the whole world during 2018 2. Breast cancer is regarded as a heterogeneous disease. Genetic and epigenetic changes have great impact on its development, progression and metastasis. Therefore, it is necessary to further clarify the mechanisms of breast cancer development and progression, which may provide a new approach to early diagnosis and prognosis prediction. Breast cancer morbidity and mortality can be significantly reduced by screening for breast cancer at an earlier and easier treatment stage 3. Currently, surgery and subsequent chemotherapy, targeted therapy or radiation therapy are the most popular way for breast cancer clinical treatment. Studies have shown that surgery combined with drug therapy can well prevent recurrence and metastasis, however, frequent drug resistance has been a major challenge in the treatment of breast cancer. Long-term continuous chemoradiotherapy can lead to certain toxic side effects and reduce patient resistance, and the effect of killing cancer cells is also poor. Breast cancer can be divided into the following categories: ductal carcinoma in situ, invasive ductal carcinoma, triple negative breast cancer (TNBC), inflammatory breast cancer, metastatic breast cancer, and other types 4. Among all breast cancer types, the TNBC subgroup is negative for estrogen receptor (ER), progesterone receptor (PR) and human epidermal growth factor receptor 2 (HER2) 5. Therefore, TNBC lacks specific therapeutic targets. In addition, TNBC is highly invasive and leads to a poor prognosis. Therefore, the management of TNBC remains a huge clinical challenge. There is an urgent need to explore predictive biomarkers and therapeutic targets for TNBC patients.

As a multifactorial disease, breast cancer is closely related to obesity and smoking 6, 7. The dual relationship between obesity and the cancer risk depends primarily on the menopause state in women. As a risk factor, the dual relationship between breast cancer and obesity can be performed in the early stages of diagnosis of obese postmenopausal women and in the early stages of diagnosis of obese premenopausal women. Accurate, painless, non-invasive and safe are vital requirement for an ideal screening method, but suffering from discomfort, radiation exposure, false positive results and overtreatment is inevitable when mammography goes 8, 9. Therefore, it is necessary to explore more accurate and sensitive biomarkers in breast cancer screening.

The development of breast cancer involves mutations in oncogenes and tumor suppressor genes, other chromosomal abnormalities are also included. Gene special deletions or copy number changes in breast cancer may change the hyperplasia to ductal carcinoma in situ. Furthermore, breast cancer may also be triggered when transcription factors are dysregulated. Series of studies have indicated that oncogenic transcription factors are related to abnormal apoptosis control and cell cycle as well as cell invasion. Recent studies have confirmed that breast cancer may also be driven by epigenetic changes, including DNA methylation, histone modifications and many others factors. Understanding the molecular mechanisms connected with breast cancer development and progression will provide strategies for identifying novel diagnostic and prognostic biomarkers while providing better prevention and treatment for breast cancer patients. Increasing evidence suggests that loss of tumor suppressor leads to increased expression of oncogenes, and increased expression of oncogenes can inhibit expression of tumor suppressor. Carcinogenesis is a multi-step phenomenon. It often appears as changes in genetic levels and controls the major intracellular pathways of cell growth and development. Increased evidence suggests that oncogenes are involved in the breast cancer introduction. Modulation of oncogenes results in a cell function that signals and contributes to a tumorigenic phenotype. At the same time, these signals produce stupendous amounts of protein, leading to cell growth and inhibition of apoptosis. The expression of specific genes is associated with estrogen receptor (ER), progesterone receptor (PR) expression and other key characteristics such as cell proliferation, invasion and metastasis 10. Therefore, specific genes have become candidate biomarkers for their pivotal role in breast cancer regulatory networks. Therefore, it is necessary to find potential biomarkers in high-risk breast cancer as a driving factor for early tumorigenesis and development.

Chromosome 19 open reading frame 48, C19orf48 is located on chromosome 19q13.33. C19orf48-encoded peptides are widely expressed in kidney tumors and other histological solid tumors 11, 12. It has been found that the donor T cell response of HLA-A*0201 restricted secondary H antigen encoded by C19orf48 may contribute to the regression of renal cell carcinoma 11. In addition, C19orf48 has been shown to be an androgen response element that may be involved in protein synthesis and trafficking, oxidative stress, transcription, proliferation, apoptosis and differentiation of prostate cancer 13. Therefore, C19orf48 may be a potential biomarker for prostate cancer. In normal breast tissue, as an age-related DNA methylation (aDNAm), C19orf48 expression is decreased, which is inversely proportional to methylation level. Because of the similar findings of breast tissue in healthy women and breast tumors, aDNAm might be a way to increase breast cancer risk with age 14.

In the present study, the mRNA expression of c19orf48 was analysed in several types of human cancers. And the relationship between the expression and survival time of breast cancer patients was explored using online database respectively. The gene co-expressed with c19orf48 was selected and enriched biology process and pathways was done.

## Material and methods

### The mRNA expression of c19orf48 in cancers based on Oncomine database

As a powerful web-based data-mining platform to analysis gene expression and DNA amplification in different cancers, Oncomine database (https://www.oncomine.org) was used to identify the expression of C19orf48 in breast cancer. Oncomine has the most comprehensive spectrum of cancer mutations, gene expression data and related clinical information, which facilitates the discovery of new biomarkers or potential therapeutic targets. In the present study, p-value<1E-4 and fold change (FC)>2 was set to do the analysis.

### The survival analysis using Kaplan-Meier plotter

The Kaplan Meier plotter (www.kmplot.com) was used to do a meta-analysis based on biomarker assessment. The effect of 54, 675 genes on survival using 18674 cancer samples including breast cancer, ovarian cancer, lung cancer, gastric cancer and pan-cancer was assessed 15. In the present study, Kaplan-Meier Survival analysis was performed to explore the relationship between the expression of c19orf48 and the survival time of different type cancers patients. The logrank p value and the hazard ratio were calculated.

### The enrichment analysis of the genes co-expressed with c19orf48

cBio Cancer Genomic portal (http://cbioportal.org) is a comprehensive online web database. Cancer genomics data including mutation, expression and copy number variance (CNV) could be explored 16. The gene co-expressed with c19orf48 in breast cancer was downloaded from cBioPortal. The genes whose absolute correlation coefficient was greater than 0.4 were selected to do the enrichment analysis using Metascape (http://metascape.org), in which the enriched ontology terms were identified using the standard accumulative hypergeometric statistical test 17. In the present study, the enriched GO biological process and KEGG pathways were selected with the threshold of p-value 0.01.^18^-^20^. At the same time, the relationship between the mRNA expression and CNV was also explored.

## Results

### c19orf48 mRNA is overexpressed in human cancers

**Figure 1:**
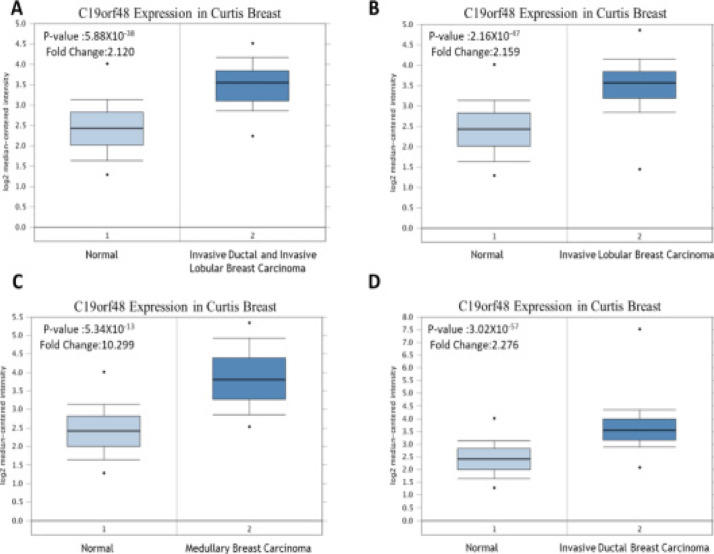
The mRNA expression of c19orf48 in breast cancers got from Oncomine database. The parameters are set as follows: FC=2, p-value=1E-4

**Figure 2:**
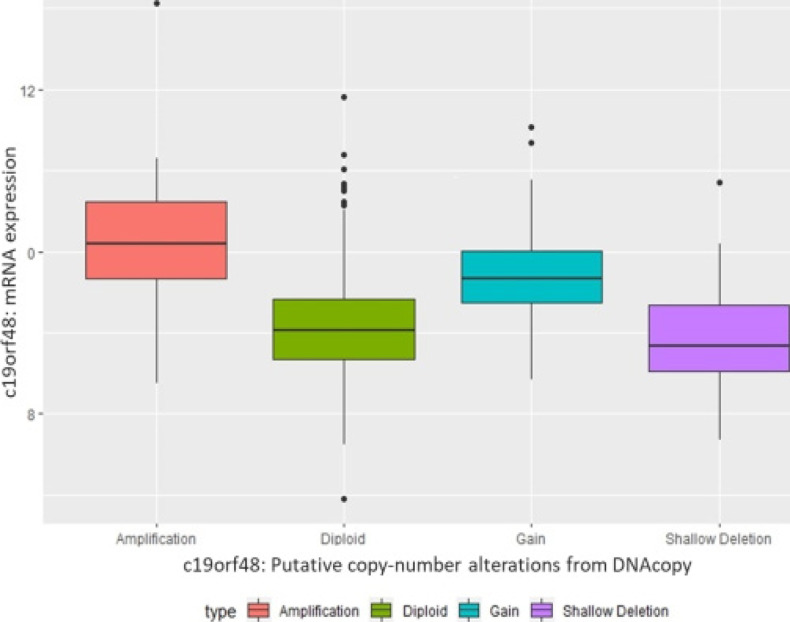
The relationship between c19orf48 mRNA expression and CNA data in breast cancer (Nature 2012 & Nat Commun 2016)

According to Oncomine results, it was found that c19orf48 was over-expressed in breast cancer compared to the normal samples (Figure 1). The relationship between CNP and mRNA expression were analysed. It was shown that the amplification of c19orf48 may contribute to the overexpression (Figure 2).

### Association of overexpressed c19orf48 with prognosis in different types of cancers

**Figure 3:**
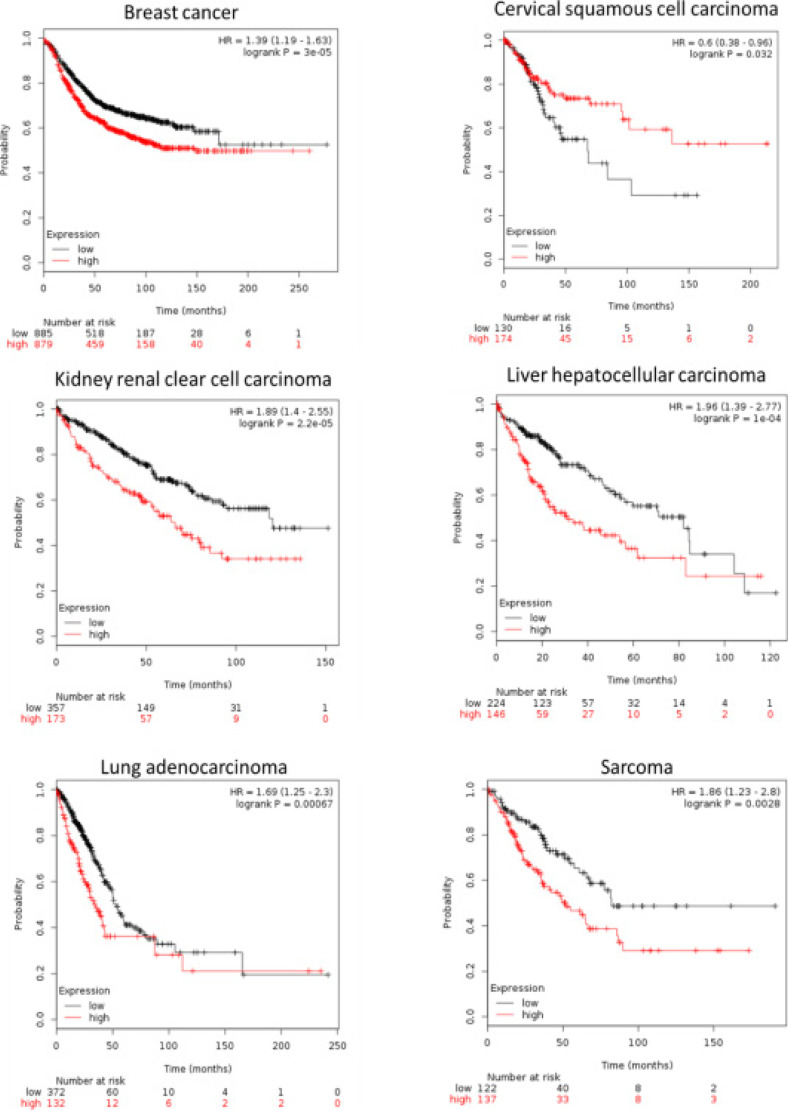
The relationship between the c19orf48 mRNA expression and the survival time of patients in several types of cancers including breast cancer(A), cervical squamous cell carcinoma(B), kidney renal clear cell carcinoma(C), liver hepatocellular carcinoma(D), lung adenocarcinoma(E) and Sarcoma(F)

The Kaplan-Meir plotter was employed to identify the relationship between the expression of c19orf48 and the overall survival time. It showed that the elevated expression of c19orf48 contributed to the short overall survival time in several types of cancers including breast cancer, cervical squamous cell carcinoma, kidney renal clear cell carcinoma, liver hepatocellular carcinoma, lung adenocarcinoma and sarcoma (Figure 3). On the basis of above finding, it could be suggested that the c19orf48 is an unfavourable prognostic biomarker in cancer development.

### Gene co-expression network analysis associated with c19orf48 in breast cancer

**Figure 4:**
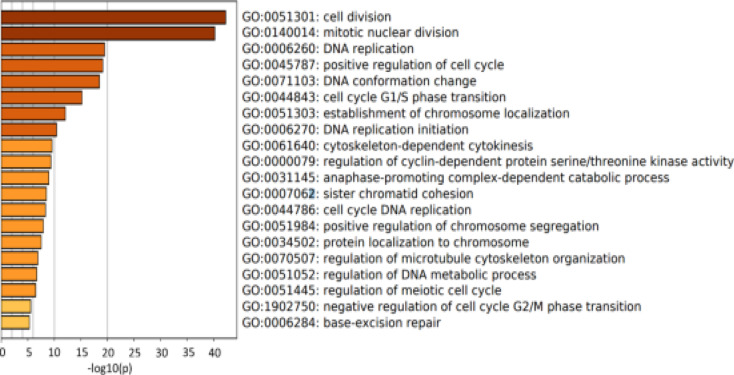
The enriched GO: BP results of the genes correlated with c19orf48 obtained by Matascape

As a relatively new gene, the detailed role of c19orf48 in breast cancer is not known yet. Protein-protein network (PPI) with the genes was constructed, which co-expressed with c19orf48 using the breast cancer dataset. In together, 141 genes correlated with c19orf48 whose absolute spearman's correlation coefficient score ≥ 0.4 were selected to construct the PPI network. It showed that the enriched GO: BP terms include cell cycle related biological process which illustrated that the potential role of c1orf48 in cell proliferation (Figure 4). The enriched KEGG pathways comprise mainly cell cycle, HTLV-infection (Table 1).

**Table 1:** The enriched KEGG results of the genes co-expressed with c19orf48 obtained by Matascape

Term ID	Description	Log(p)
hsa04110	Cell cycle	-27.611
hsa04114	Oocyte meiosis	-8.51
hsa05166	HTLV-I infection	-7.432
hsa03030	DNA replication	-7.222
hsa05222	Small cell lung cancer	-2.825
hsa03440	Homologous recombination	-2.753
hsa03010	Ribosome	-2.672

## Discussion

Breast cancer poses a great threat to the health of the world and is one of cancer death reasons among women 21. Breast cancer mortality has declined due to early detection and advanced treatment. However, the complex mechanisms of breast cancer development and progression remain a significant barrier to the treatment of this disease. At present, effective tools for evaluating the treatment effects and predicting prognosis in breast cancer patients are always wanted. The TNM staging system can accurately assess tumor size, lymphatic involvement and distant metastasis 22, 23. A more precise system for indicating the molecular characteristics of breast cancer is needed for personalization and precise treatment. The study of breast cancer biomarkers will fill this gap. Early diagnosis is critical for the prognosis. However, the median size of clinically diagnosed breast tumors is currently 2 to 2.5 cm, which may be advanced (stage III) breast tumors that have metastasized from tissue to axillary lymph nodes. At present, there is a lack of highly accurate breast cancer diagnostic tests e.g., the sensitivity of standard mammography tests is only 54% to 77%. Ultrasound, computed tomography (CT) and magnetic resonance imaging (MRI) have higher sensitivity but higher cost 24. Therefore, a more precise, cost-effective and non-invasive alternatives for diagnosis in breast cancer is what urgently needed currently.

In oncology, biomarkers are regarded can be used to predict the malignant potential, prognosis, or response to treatment of a tumor. Therefore, a more effective marker is urgently needed. Conventional histopathology is difficult to predict the breast cancer patient prognosis. Therefore, biomarkers indicating intrinsic features of tumors at the molecular level have become a hot spot in biomedical research. Many biomarkers are widely used in current clinical practice 25. For example, hormone receptors for breast cancer subtype classification and genes associated with genome maintenance can be used to predict breast cancer susceptibility 26. Estrogen receptor (ER), progesterone receptor (PgR) and human epidermal growth factor receptor 2 (HER2) have been identified as prognostic biomarkers and therapeutic targets for breast cancer 27, 28. However, many biomarkers require further investigation in order to find better biomarkers and drug development goals 29. In addition, many single- or multi-gene biomarkers have been allowed in clinical applications of breast cancer patient prognosis and to promote the breast cancer diagnosis and management, especially in the early detection, accurate prediction of metastasis and treatment options. However, most of them are still in the preclinical stage. Therefore, it is necessary to explore more sensitive markers in clinical development to improve the breast cancer patient diagnosis, prognosis and therapy. Despite significant advances in the prevention, diagnosis, and therapy, metastatic breast cancer is still a disease can't be cure. At the molecular level, breast cancer, one of the heterogeneous diseases, whose development and progression is regulated by genes connected with cell growth, proliferation and differentiation. With the advent of the era of targeted therapy, increasing molecularly targeted drugs are available for clinical practice. However, the main challenge currently remains to identify predictive biomarkers for selecting the best treatment to protect patients with breast cancer from treatment-related side effects and to minimize treatment costs.

In the present study, employed the Oncomine database to identify the expression of C19orf48 in different type of cancers was analysed. Oncomine has the most comprehensive spectrum of cancer mutations, gene expression data and related clinical information, which facilitates the discovery of new biomarkers or potential therapeutic targets. In addition, cBioPortal integrates data from 126 oncology genome research projects, including TCGA, the International Cancer Genome Consortium and other large oncology research projects, in addition, it includes data from 28,000 cases 30. In recent years, ever-increasing amounts and quality of data have caused to the unit of biological networks, with the final goal of revealing potential cellular processes 30. Therefore, protein-protein interactions (PPIs) have become one of the most vital and researched networks. The PPI network is a useful tool for understanding cell function, disease mechanisms, and drug design or repositioning. It helps to study the molecular mechanisms of disease from a systems perspective and to discover new drug targets.

C19orf48 encodes a minor histocompatibility antigen. In renal cell carcinoma patients, this antigen is found being identified by CD8+ cytotoxic T cells. After MHC-matched allergic nonogeneloablative allogeneic hematopoietic cell transplantation (HCT), induce T cell responses to HLA-A*0201 restricted secondary H antigen encoded by C19orf48 may contribute to regression of renal cell carcinoma. C19orf48 is up-regulated in prostate cancer. Prostate development and maintenance are dependent on androgen and androgen receptors. The androgen pathway remains important in prostate cancer. One study evaluated the transcriptome results of androgen-resistant prostate cancer cells using a long sequence analysis (Long SAGE) library of gene expression showed that C19orf48 is up-regulated in the androgen response. The research indicated that C19orf48 is vital in cell-cycle related processes.

Breast cancer incidence is positively correlated with age, and breast cancer gene methylation is also associated with aging. The research on aDNAm in women's normal breast tissue without evidence of cancer or breast benign disease can provide insight into the naturally occurring conditions in the breast and promote the treatment of breast cancer. Previous study identified 1,214 aDNAm in all autosomes, most of which are methylated and associated with increased age, and are commonly detected in CpG islands and non-enhancers. Among them, there is an obvious negative connection between C19orf48 gene expression and methylation level. Furthermore, based on the independent TCGA-BRCA dataset, compared to adjacent normal tissue and normal breast tissue, C19orf48 verified in women breast tissue without breast cancer history is highly methylated in breast tumors. Compared to other subtypes, C19orf48 has a lower level of methylation in basal tumors 14. Therefore, C19orf48 could contribute to the development of breast cancer.

In conclusion, c19orf48 may be a useful and predictive biomarker for the prognosis of breast cancer patients. To the best of our knowledge, this is the first report describing the expression of c19orf48, the potential driving factor led to this and its effect.
